# Myocardial native T1 and extracellular volume with healthy ageing and gender

**DOI:** 10.1093/ehjci/jey034

**Published:** 2018-03-30

**Authors:** Stefania Rosmini, Heerajnarain Bulluck, Gabriella Captur, Thomas A Treibel, Amna Abdel-Gadir, Anish N Bhuva, Veronica Culotta, Ahmed Merghani, Marianna Fontana, Viviana Maestrini, Anna S Herrey, Kelvin Chow, Richard B Thompson, Stefan K Piechnik, Peter Kellman, Charlotte Manisty, James C Moon

**Affiliations:** 1 Barts Heart Centre, St. Bartholomew’s Hospital, London, UK; 2 Institute of Cardiovascular Science, University College, West Smithfield, London EC1A 7BE, UK; 3 Department of Cardiovascular Sciences, St Georges, University of London, London, UK; 4 National Amyloidosis Centre, Royal Free Hospital, London, UK; 5 Department of Cardiovascular, Respiratory, Nephrology, Anaesthesiology, and Geriatric Sciences, “Sapienza” University of Rome, Rome, Italy; 6 Department of Biomedical Engineering, Faculty of Medicine and Dentistry, University of Alberta, Edmonton, Canada; 7 Division of Cardiovascular Medicine, Radcliffe Department of Medicine, University of Oxford, Oxford, UK; 8 National Heart, Lung and Blood Institute, National Institutes of Health, Bethesda, USA

**Keywords:** T1 mapping, myocardial T1, extracellular volume, age, gender, healthy volunteers

## Abstract

**Aims:**

To determine how native myocardial T1 and extracellular volume (ECV) change with age, both to understand aging and to inform on normal reference ranges.

**Methods and results:**

Ninety-four healthy volunteers with no a history or symptoms of cardiovascular disease or diabetes underwent cardiovascular magnetic resonance at 1.5 T. Mid-ventricular short axis native and post-contrast T1 maps by Shortened MOdified Look-Locker Inversion-recovery (ShMOLLI), MOdified Look-Locker Inversion Recovery (MOLLI) [pre-contrast: 5s(3s)3s, post-contrast: 4s(1s)3s(1s)2s] and saturation recovery single-shot acquisition (SASHA) were acquired and ECV by these three techniques were derived for the mid anteroseptum. Mean age was 50 ± 14 years (range 20–76), male 52%, with no age difference between genders (males 51 ± 14 years; females 49 ± 15 years, *P* = 0.55). Quoting respectively ShMOLLI, MOLLI, SASHA throughout, mean myocardial T1 was 957 ± 30 ms, 1025 ± 38 ms, 1144 ± 45 ms (*P* < 0.0001) and ECV 28.4 ± 3.0% [95% confidence interval (CI) 27.8–29.0], 27.3 ± 2.7 (95% CI 26.8–27.9), 24.1 ± 2.9% (95% CI 23.5–24.7) (*P* < 0.0001), with all values higher in females for all techniques (T1 +18 ms, +35 ms, +51 ms; ECV +2.7%, +2.6%, +3.4%). Native myocardial T1 reduced slightly with age (*R*^2^ = 0.042, *P* = 0.048; *R*^2^ = 0.131, *P* < 0.0001—on average by 8–11 ms/decade—but not for SASHA (*R*^2^ = 0.033 and *P* = 0.083). ECV did not change with age (*R*^2^ = 0.003, *P* = 0.582; *R*^2^ = 0.002, *P* = 0.689; *R*^2^ = 0.003, *P* = 0.615). Heart rate decreased slightly with age (*R*^2^ = 0.075, coefficient = −0.273, *P* = 0.008), but there was no relationship between age and other blood T1 influences (haematocrit, iron, high density lipoprotein-cholesterol).

**Conclusion:**

Gender influences native T1 and ECV with women having a higher native T1 and ECV. Native T1 measured by MOLLI and ShMOLLI was slightly lower with increasing age but not with SASHA and ECV was independent of age for all techniques.

## Introduction

Measurement of native T1 and extracellular volume (ECV) by cardiovascular magnetic resonance (CMR) allow quantification of diffuse myocardial fibrosis. The evidence is conflicting as to whether myocardial fibrosis increases with age with some studies suggesting it decreses^1^ and others pointing to different age related processes, e.g. myocardial lipofuschin or haemosiderin accumulation.^2–4^ Whether the ECV increases with age is unclear. The Multi-Ethnic Study of Atherosclerosis found a tiny correlation of ECV with age (*R*^2^ = 0.021, *P* = 0.012)^5^ but used region of interest (ROI) based on measurement rather than mapping, and studied a population with high rates of diabetes and hypertension. Others found no changes^6^^,^^7^ or found increases but with small populations^8^ or with significant comorbidities.^9^^,^^10^ Using best available mapping techniques and a cohort designed specifically to address healthy ageing curated by excluding established confounding comorbidities or cardiac disease, we sought to determine whether T1 and ECV increase with age—both in order to understand the aging biology and as a step towards developing normal reference ranges.

## Methods

The study comply with the Declaration of Helsinki and obtained approval from the local research ethics committee. Healthy volunteers were recruited through advertising in the hospital. Pre-consent, we excluded patients with a history or symptoms of cardiovascular disease or diabetes. All participants provided written informed consent. A stratified approach was adopted for recruitment to ensure adequate representation of participants in each age decile. A blood sample was drawn from each subject the same day just before (approximately 30 min) the CMR examination to acquire the main biochemical parameters and in particular haematocrit (Hct) for ECV quantification.

The scans were performed at 1.5-T (Magnetom Avanto; Siemens Medical Solutions, Erlangen, Germany) with 32-channel cardiac phased array receiver. The imaging protocol included cines, native T1 mapping, T2 mapping, late gadolinium enhancement, and post-contrast T1 mapping.

### T1 mapping

T1 maps were acquired using three different sequences: Shortened Modified Look-Locker Inversion recovery (ShMOLLI),^11^ a MOdified Look-Locker Inversion Recovery (MOLLI),^12^ and a saturation recovery single-shot acquisition (SASHA)^13^ pre and at approximately 15 min after the injection of 0.1 mmol/kg of Gadoterate meglumine (Gd-DOTA marketed as Dotarem, Guerbet S.A., Paris, France).

For the ShMOLLI technique, pre- and post-contrast T1 maps were generated inline by merging images from three consecutive inversion recovery experiments as previously described.^11^ The typical acquisition parameters were: echo time = 1.05 ms; imaging duration = 210 ms; matrix = 192 × 140; phase partial Fourier 6/8; minimum TI= 110 ms; TI increment = 80 ms; flip angle 35°; slice thickness = 8 mm.

For the MOLLI technique (work-in-progress 448b), sampling was in seconds and optimised for expected measured T1s [pre-contrast 5s(3s)3s, post-contrast 4s(1s)3s(1s)2s]^14^ with shortened inversion pulse for improved efficiency and reduced T2 dependence.^15^ The acquisition parameters were: pixel bandwidth 977 Hz/pixel; echo time = 1.14 ms; flip angle = 35°; matrix = 256 × 144; slice thickness = 6 mm. Inline motion correction and a non-linear least-square curve fitting were performed with the set of images acquired at different inversion times to generate a pixel-wise coloured T1 map.

For the SASHA technique,^11^ the acquisition parameters for pre and post contrast maps were: echo time = 1.36 ms; matrix = 256 × 149; flip angle = 70°; slice thickness = 8 mm, and with a variable flip angle readout. Saturation recovery images were acquired and reconstructed as previously described using a two parameter model^12^ with motion correction to generate pixel-wise coloured T1 map from the scanner.^13^

Further details of the various T1 acquisition parameters are available from the online appendix. A four chamber and a mid ventricular short axis slice were acquired for all the three T1 mapping sequences but only the latter was used for all analysis.

### MOLLI ECV maps

The previously described and validated automated method for producing a pixel-wise ECV map was used for MOLLI T1 mapping.^16^ This method corrects for respiratory motion due to variation in breath-holding as well as patient movement between breath-holds and relies on co-registration of the native and post-contrast T1 pixel maps. An offline software (ECV Mapping Tool Version 1.1) subsequently generated pixel-wise ECV maps after adjusting for Hct using a variety of post processing steps as previously described.^16^

### T1 mapping analysis and ECV quantification

**Figure 1:**
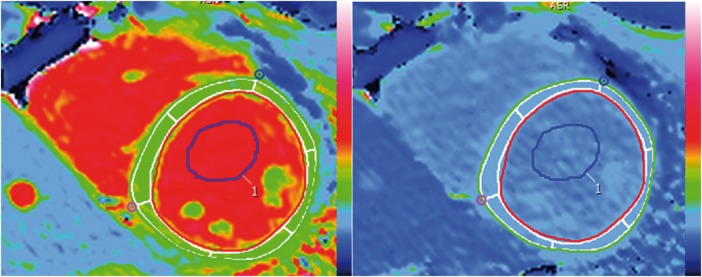
Example of myocardial and blood ROIs in ShMOLLI native (left) and post-contrast (right) T1 maps. Manual epicardial (green) and endocardial (red) contours with 10% erosion offset (white borders) drawn in the native ShMOLLI T1 map (left) and exported on to the post-contrast map (right) are shown. The myocardial ROI is segmented according to the AHA model. A blood ROI was also drawn in the native T1 map and copied on to the post-contrast map.

All T1 maps were analysed using cvi^42^ software (Circle Cardiovascular Imaging Inc., Version 5.1.2[303], Calgary, Canada). For myocardial T1 analysis, manual epicardial and endocardial contours were drawn on the MOLLI mid ventricular short axis slice and segmented according to the American Heart Association (AHA) segmentation. The mid antero-septum (segment 8) was used for the analysis. In order to avoid confounders, with blood partial voluming being a particular concern, the effect of different degrees of endocardial and epicardial border erosion were initially assessed. The initial contours were drawn on what was visually considered to be the endocardial and epicardial boundaries on the maps and subsequently an off-set erosion of 10%, 20%, and 30% was applied on both the endocardial and epicardial borders using a function provided by the software as illustrated in *Figure 1*. With initial erosion (0–10%), measured myocardial T1 pre-contrast was lower and post-contrast increased suggesting partial voluming was being reduced. Above 10%, this reduction stopped (e.g. MOLLI: 10%, 20%, 30% erosion: native T1 1028 ms, 1019 ms, 1014 ms) so (given a predilection for fibrosis to be endocardial), a 10% erosion offset was used. These endocardial and epicardial borders were then copied on to the pre-contrast ShMOLLI and SASHA T1 maps and MOLLI ECV map and ShMOLLI and SASHA post-contrast T1 maps with manual adjustment if needed. An example of the myocardial and blood ROI is provided in *Figure 1*.

For blood analysis, a ROI was drawn in the left ventricular (LV) blood pool of the mid ventricular short axis slice of the pre-contrast MOLLI T1 map and care was taken to avoid papillary muscles. This ROI was then copied on to the native ShMOLLI and SASHA T1 maps. Very limited adjustments were needed due to changes in cardiac position, movement/different breath-holds and slightly smaller cavity in some ShMOLLI images.

Extracellular volume was calculated using the mean segmental pixel value from the MOLLI ECV maps and using the formula ECV =  (Δ[1/T1myo]/Δ[1/T1blood]) * [1-Hct]) for ShMOLLI and SASHA.^17^

### Statistical analysis

Statistical analysis was performed using R (version 3.0.1, 2013) and SPSS (Version 22, IBM Corporation, IL, USA). *A priori*, we set out to define normal values for native T1 and ECV in health by MOLLI, ShMOLLI, and SASHA as the range of values containing the central 95% (2 SD) of native T1 and ECV readings in the ‘healthy’ population, with reference limits of 2.5% and 97.5%. This definition results in 5% of the ‘healthy’ population being classified as ‘abnormal’ or as high native T1/ECV candidates. The study was therefore designed to enrol a representative group of healthy volunteers which would allow at least two subjects (1 male, 1 female) for each 2.5% interval of native T1/ECV for testing that is *n* = 80. *A priori*, we inflated recruitment to 105 to account for potential participant exclusion (in fact, we excluded 11). Our final reference group of *n* = 94 thus allowed us close to three subjects for testing across the majority of 2.5% native T1/ECV intervals for understanding normal variation in health.

Shapiro–Wilk test was used to assess for normality. Normally distributed continuous variables were presented as mean ± standard deviation. Categorical data were reported as frequencies and percentages. A two-sample independent *t*-test was used to compared normally distributed continuous variables.

To adjust for clustering among and by groups, a varying intercept model was fitted using R package ‘lme4’ to provide a mixed-effect modelling framework for studying the impact of age and gender on group-level variation in native T1 and ECV in study subjects. For each of T1 and ECV we compared the full model (with the fixed effects of age and gender) against a reduced model without the fixed effects to determine whether the fixed effects were significant based on the difference between the likelihood of these two models by conducting the likelihood ratio test using the ANOVA function to determine χ^2^ values.

All statistical tests were two-tailed, and *P*-values of less than 0.05 were considered statistically significant.

## Results

**Figure 2:**
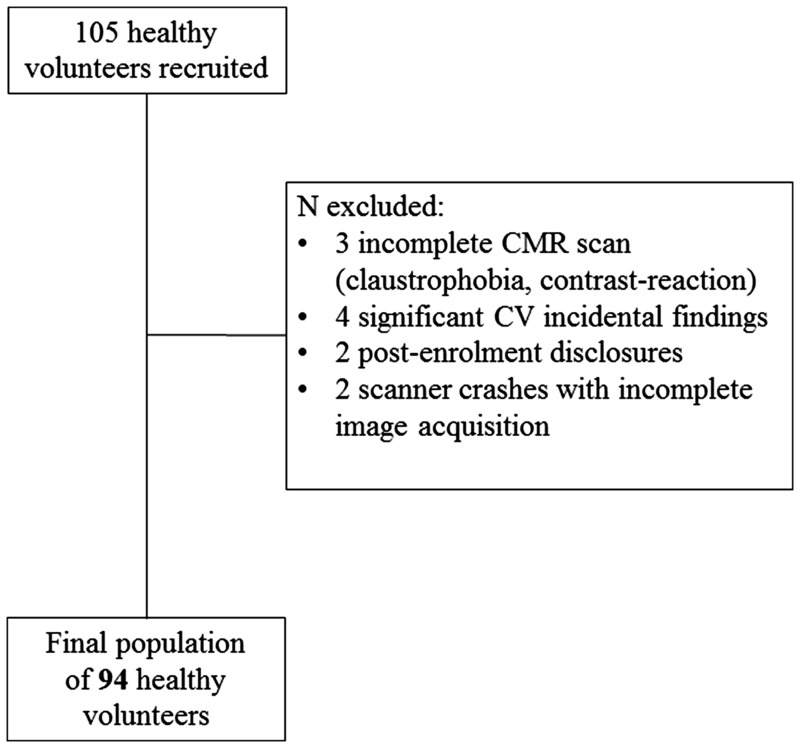
Population selection process. In the flow chart is illustrated the final population selection process. CV, cardiovascular.

One hundred and five healthy volunteers were recruited and underwent blood tests, 12-lead electrocardiogram (ECG) and CMR. Any subject found with abnormalities on ECG or CMR (with the exception of inconsequential extra-cardiac findings such as liver cysts which were allowed) was excluded. There were 11 such exclusions: 3 not completing the CMR (1 contrast reaction resulting in premature termination of the scan, 2 for claustrophobia); 4 for incidental findings (1 CMR finding of pulmonary valve stenosis, 1 CMR finding of biventricular impairment, 1 blood test high glucose—subsequently confirmed diabetes, 1 high blood pressure and ECG criteria for LV hypertrophy); 2 for post-enrolment disclosures (1 on beta-blockers therapy for atrial ectopics, 1 due to previous breast cancer treatment including left sided radiotherapy), and 2 due to scanner crashes with post-contrast images not available. The result was a final study population of 94 volunteers (*Figure 2*). As expected, these had some cardiovascular risk factors: smoking [1 (1%) active, 17 (18% ex-smokers); hypertension [none diagnosed; 7 (7%) had blood pressure >140/90 mmHg on attendance); family history: 1 (1%) of coronary artery disease in a first degree relative (mother suffering a myocardial infarction aged 64), 1 (1%) of premature sudden cardiac death <40 year old in first degree relative. Five (5%) had hypercholesterolemia and were on statins for primary prevention and 15 (16%) had a total cholesterol (here unfasted) above 6.2 mmol/L (240 mg/dL). The average body mass index (BMI) was 24.4 ± 3.7 with nine volunteers having BMI > 30 with one having the highest value of 36. No one had peripheral vascular disease. Ethnicity was 68 (72%), Caucasian, 14 (15%) Asian, 7 (7%) Black/Afro-caribbean, 5 (5%) other. Six (6%) had medications for primary prevention (five on statins, one on aspirin). All volunteers had a normal 12-lead ECG and all the CMR scans were reported as normal by experienced Level 3 CMR physicians. Mean age was 50 ± 14 years, range 20–76 years, 52% male. The age decile distribution was: 20–29 (*n* = 10), 30–39 (*n* = 14), 40–49 (*n* = 24), 50–59 (*n* = 21), 60–69 (*n* = 16), and 70–79 (*n* = 9). Clinical characteristics of the overall population and according to gender are described in *Table 1*. There were no gender differences in age (males 51 ± 14 years and females 49 ± 15 years, *P* = 0.55). Heart rate decreased slightly with age (*R*^2^ = 0.075, *P* = 0.008) (*Figure 2*, top right). There was no relationship between age and other blood variables that have influences on blood T1, in particular Hct (*R*^2^ = 0.008, *P* = 0.402), iron bound to transferrin (*R*^2^ = 0.003, *P* = 0.617) and high density lipoprotein-cholesterol (*R*^2^ = 0.020, *P* = 0.182) (Supplementary data online, *Figure S1*, top left and bottom right and left).

**Table 1:** Clinical characteristics of the 94 healthy volunteers

	Overall population (*n*_** **_=_** **_94)	Males (*n*_** **_=_** **_49, 52%)	Females (*n*_** **_=_** **_45, 48%)
Age (years)	50 ± 14	51 ± 14	49 ± 15
SBP (mmHg)	122 ± 13	125 ± 12	120 ± 13
DBP (mmHg)	76 ± 9	77 ± 7	75 ± 10
EDV (mL)	132 ± 32	149 ± 34	115 ± 19
ESV (mL)	44 ± 13	51 ± 14	37 ± 9
LV mass (g)	123 ± 34	144 ± 30	100 ± 20
LVSV (mL)	88 ± 21	97 ± 24	77 ± 12
LVEF (%)	67 ± 4	66 ± 4	68 ± 4
LAAi (cm^2^/m^2^)	11 ± 2	11 ± 2	11 ± 2
Hct (L/L)	0.42 ± 0.04	0.44 ± 0.03	0.39 ± 0.03

Data reported as mean ± SD.

DBP, diastolic blood pressure; EDV, end-diastolic volume; ESV, end-systolic volume; Hct, haematocrit; LAAi, left atrial area indexed; LVEF, left ventricular ejection fraction; SBP, systolic blood pressure; SD: standard deviation; LVSV, left ventricular stroke volume.

Acquisition of the mapping sequences was particularly careful in the context of the research project and the need for repeat acquisition was limited to <5% of healthy volunteers. This was typically one additional breath-hold per slice, although there were two outlier subjects where multiple breath-holds were needed. There was one power-cut (requiring restart) and one period where the SASHA sequence did not work for post-contrast acquisitions. Artefacts were typically breathing artefacts for ShMOLLI (a non-MOCO sequence, albeit shorter breath-hold) with misgating more common in MOLLI and SASHA. In three subjects, the source images were good, but the ECV map failed—these required post-acquisition individualised post-processing (author P.K.) for reconstruction.

Considering all study subjects of all ages and across both genders, overall mean values of native T1 and ECV (reported in the order of ShMOLLI, MOLLI, and SASHA, respectively throughout the manuscript) were T1: 957 ± 30 ms, 1025 ± 38 ms, 1144 ± 45 ms and ECV: 28.4 ± 3.0% [95% confidence interval (CI) 27.8–29.0], 27.3 ± 2.7 (95% CI 26.8–27.9), 24.1 ± 2.9% (95% CI 23.5–24.7), *Table 2*.

**Table 2:** T1 mapping data in the overall population of 94 healthy volunteers.

	Overall (*n*_** **_=_** **_94)	Males (*n*_** **_=_** **_49, 52%)	Females (*n*_** **_=_** **_45, 48%)	*P*-value
Myocardial native T1					
ShMOLLI (ms)	957 ± 30	948 ± 26	966 ± 31	0.003
MOLLI (ms)	1024 ± 39	1008 ± 33	1043 ± 37	<0.0001
SASHA (ms)	1144 ± 45	1120 ± 35	1171 ± 41	<0.0001
ECV					
ShMOLLI (%)	28.4 ± 3.0	27.1 ± 2.7	29.8 ± 2.7	<0.0001
MOLLI (%)	27.3 ± 2.7	26.1 ± 2.3	28.7 ± 2.6	<0.0001
SASHA (%)	24.1 ± 2.9	22.6 ± 2.3	26.0 ± 2.4	<0.0001

ECV, extra-cellular volume; MOLLI, MOdified Look-Locker Inversion recovery; ShMOLLI, Shortened MOdified Look-Locker Inversion recovery; SASHA, saturation recovery single-shot acquisition.

Gender: For both native T1 and ECV, females had higher values by all techniques (T1 +18 ms, +35 ms, +51 ms; ECV +2.7%, +2.6%, +3.4%, *P*-value being statistically significant for all parameters), *Table 2*.

**Figure 3:**
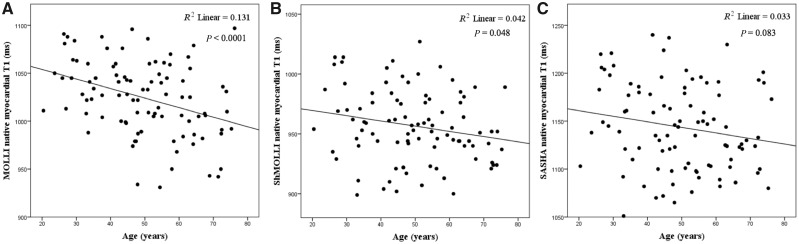
Relationship between age and native myocardial T1 according to MOLLI, ShMOLLI and SASHA. Native myocardial T1 decreased slightly with age by (*A*) MOLLI (*R*^2^ = 0.131, *P* < 0.0001) and (*B*) ShMOLLI (*R*^2^ = 0.042, *P* = 0.048), while this was not the case for (*C*) SASHA (*R*^2^ = 0.033, *P* = 0.083).

**Figure 4:**
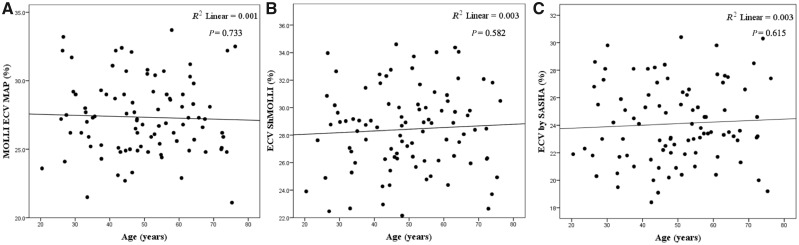
Relationship between ECV and age according to MOLLI, ShMOLLI and SASHA. *R*^2^ = 0.001, *P* = 0.733 by MOLLI (*A*), *R*^2^ = 0.003, *P* = 0.582 by ShMOLLI (*B*) and *R*^2^ = 0.003, *P* = 0.615 by SASHA (*C*).

Age: native myocardial T1 was slightly lower with increasing age (*R*^2^ = 0.042, *P* = 0.048; *R*^2^ = 0.131, *P* < 0.0001), on average by 8 and 11 ms/decade by ShMOLLI and MOLLI but not by SASHA (*R*^2^ = 0.033, *P* = 0.083) (*Figure 3*). This was in both males (*R*^2^ = 0.130, *P* = 0.011) and females (*R*^2^ = 0.150, *P* = 0.009). ECV did not change significantly with age by any technique: (*R*^2^ = 0.003, *P* = 0.582; *R*^2^ = 0.001, *P* = 0.733 and *R*^2^ = 0.003, *P* = 0.615, *Figure 4*).

Adjusting for clustered data, analysis for T1 by the three sequences using linear models with age and gender as fixed effects, confirmed how female gender affected T1 (χ2 (1)= 62.87, *P* =<0.0001), increasing it by about 33 ms and how increasing age also affected T1 (χ^2^ (1)= 25.62, *P* = 0.0001), decreasing it by 28, 25, 26, 33, and 41 ms across 2nd, 3rd, 4th, 5th, and 6th decades, respectively.

Similar analysis for ECV by the three sequences confirmed how female gender affected ECV (χ^2^ (1)= 82.07, *P* = <0.0001), increasing it by about 2.9 ECV points and how increasing age did not significantly affect ECV (χ^2^ (1)= 10.98, *P* = 0.05), describing small changes of the order −0.83, −0.13, 0.28, 0.62 and −0.79 ECV points across 2nd, 3rd, 4th, 5th, and 6th decades, respectively.

## Discussion

Native myocardial T1 and ECV allow quantification of myocardial fibrosis and are increasingly used in the clinical practice for the diagnosis of several cardiac disorders, such as cardiomyopathies, myocarditis, iron overload and ischaemic heart disease,^18^^,^^19^ so the need for accurately establishing normal reference ranges and understanding variations with physiological parameters is clear. We, therefore, prospectively aimed to understand changes in these parameters with healthy aging and gender, using best available technology including three T1 mapping sequences and two ECV analysis techniques. We found that native T1 and ECV were higher in females, as previously found^20^^,^^21^ but, that native T1 was slight lower with age (ShMOLLI and MOLLI), and ECV does not change. The known measurement differences by ShMOLLI, MOLLI, and SASHA were present for T1 and although reduced for ECV were still present. The meticulous selection of the cohort to be healthy, its size, the prospective nature of the study, the way blood dependent variables were checked as confounders, the use of three sequences and their quality control by the physicists that wrote them and the use of two analysis techniques (both drawn regions of interest and ECV mapping), are strengths of the study and suggest the results are robust.

There is a belief that myocardial fibrosis increases with age. There is little supporting evidence and some studies say the opposite with an increase in myocyte size and volume fraction with a decrease in the relative amount of interstitium.^1^ This study explored healthy aging—our subjects had no comorbidities (no diabetes, no hypertension), both of which increased in prevalence with age.

A recent group similarly showed in a smaller cohort of 44 individuals free from cardiovascular disease, diabetes, hypertension, and not on any cardiovascular medication, that native myocardial T1 measured by SASHA was higher in women compared to men and did not vary significantly with age (*P* = 0.59) while ECV did not vary significantly with age (*P* = 0.20) or gender (*P* = 0.14).^22^

Other authors point to other age related changes e.g. myocardial lipofuschin or haemosiderin accumulation.^2–4^ These could well account for the native T1 being lower with age with constant ECV. The native T1 technique measures signal from both myocytes and interstitum, and the ECV measurement includes plasma volume. There are plausible reasons that, for example capilliary density or resting recruitment could be different with age, but this is not convincing as vasodilatation would increase both native T1 and ECV. Little data is available in this area on healthy ageing.^23^^,^^24^ The gender differences found are similar to all other literature. Partial voluming of blood pool has always been a plausible reason for this with the thinner (on average) female heart more predisposed to this. This, and the longer blood T1 in capilliaries within myocardium^25^ remain plausible but look increasingly unlikely to be the whole explanation.

As with other studies (e.g. reference ranges for volume analysis), the data here is described as being related to ageing. This is not strictly true—they are all single timepoint study of different birth-cohorts. There is the potential that 60 and 70 year olds, whose fetal development and early growth occurred in the immediate post war period could be different to that of 20–30 year olds. Long term follow-up studies with paired data decades apart are extraordinarily difficult to do as the technology changes so fast. These will however be needed in the future.

We used three sequences: one Saturation recovery based (SASHA) and two inversion recovery based sequences (ShMOLLI and MOLLI). We did this for generalizability of results but also because the accuracy and precision of the sequences are not known, particularly when applied *in vivo*. There are also differences in what is being measured—in particular SASHA is less magnetization transfer sensitive than infrared based sequences. We were looking for the biology of healthy ageing. Trends (or lack of trends) appearing in all three sequences would therefore be robust.

### Limitations

The slice thickness for MOLLI was 6 mm (Avanto 6 mm by Siemens) compared to 8 mm for ShMOLLI and SASHA. Corresponding automated ECV map for ShMOLLI and SASHA were not available at the time of this study. We adopted a stratified approach to recruiting patients but this was done to ensure satisfactory distribution of participants in each age decile.

## Conclusion

In health, gender influences native myocardial T1 and ECV with women having a higher native T1 and ECV compared with males. Native myocardial T1 is slightly lower with increasing age while ECV is not influenced by aging.

## Supplementary data

Supplementary data are available at *European Heart Journal - Cardiovascular Imaging* online.

**Conflict of interest:** None declared.

## Funding

S.R. is supported by Borse di studio SIC e MSD Italia-Merck Sharp & Dohme. M.F is supported by Clinical Research Training Fellowship from the British Heart Foundation (FS/12/56/29723). T.A.T is supported by Doctoral Research Fellowship from the NIHR, UK (NIHR-DRF-2013-06-102). A.A.G. is supported by the Rosetrees Trust. J.C.M. has received grant funding from GlaxoSmithKline. This work was undertaken at University College London Hospital, which received a proportion of funding from the UK Department of Health National Institute for Health Research Biomedical Research Centers funding scheme. SKP is supported by the National Institute for Health Research (NIHR) Oxford Biomedical Research Centre based at The Oxford University Hospitals Trust at the University of Oxford. The views expressed are those of the author(s) and not necessarily those of the NHS, the NIHR or the Department of Health.

## Supplementary Material

Supplementary Figure 1S

Supplementary Data
