# Supplementary figures and images for: Myocardial native T1 and extracellular volume with healthy ageing and gender
Source: Eur Heart J Cardiovasc Imaging. 2018 Mar 30;19(6):615–21. doi: 10.1093/ehjci/jey034 (PMC5963299; doi:10.1093/ehjci/jey034)

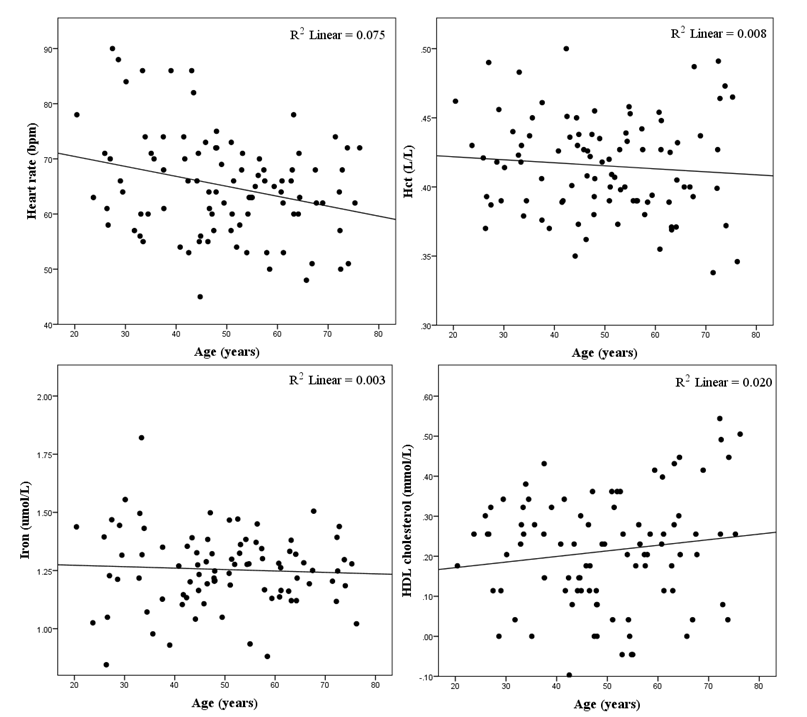

Supplement: Supplementary Figure 1S [file jey034_figure_1s.png]
