# Supplementary material for: Myocardial native T1 and extracellular volume with healthy ageing and gender
Source: Eur Heart J Cardiovasc Imaging. 2018 Mar 30;19(6):615–21. doi: 10.1093/ehjci/jey034 (PMC5963299; doi:10.1093/ehjci/jey034)

SUPPLEMENTARY DATA

***Native myocardial T1 and extracellular volume age and gender-related variations in health***

*Rosmini S et al.*

**Sequence parameters**

All the scans were performed on a 1.5 T Siemens Avanto scanner (Siemens Medical Solutions, Erlangen, Germany; system software version VB17) using a 32-channel cardiac coil array. MOLLI, ShMOLLI and SASHA variant were run for the cohort of healthy volunteers.

Complete sequence details can be found on the following pages as detailed below:

1. Pages 2 & 3 – pre-contrast 5s(3s)3s MOLLI indicating 2 inversions with acquisition of images for at least 5 seconds, followed by a recovery of at least 3 seconds, and a second inversion with images acquired for at least 3 seconds.
2. Pages 4 & 5 – post-contrast 4s(1s)3s(1s)2s MOLLI indicating 3 inversions with acquisition of images for at least 4 seconds, followed by a recovery of at least 1 second, a second inversion with images acquired for at least 3 seconds, followed by a recovery of at least 1 second, and a final acquisition of 2 seconds.
3. Pages 6 & 7 - ShMOLLI.
4. Pages 8 & 9 – SASHA.


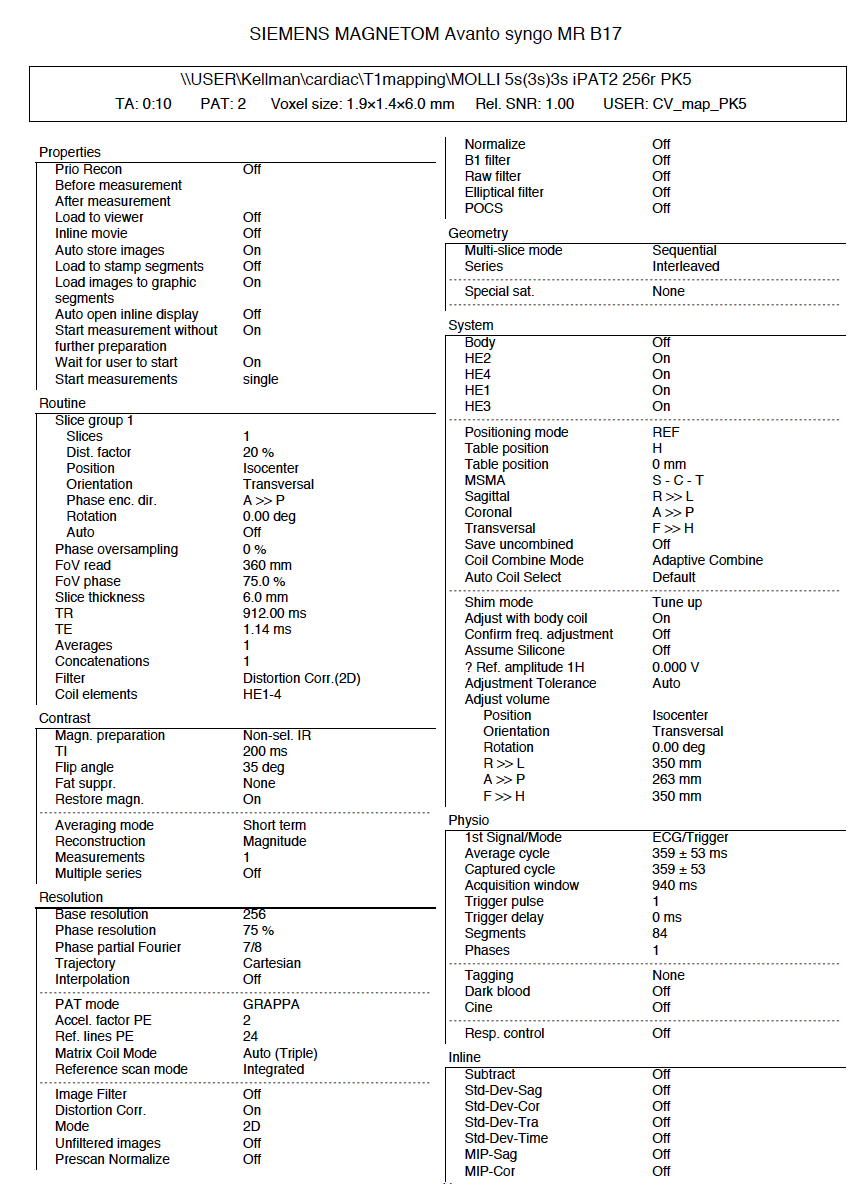


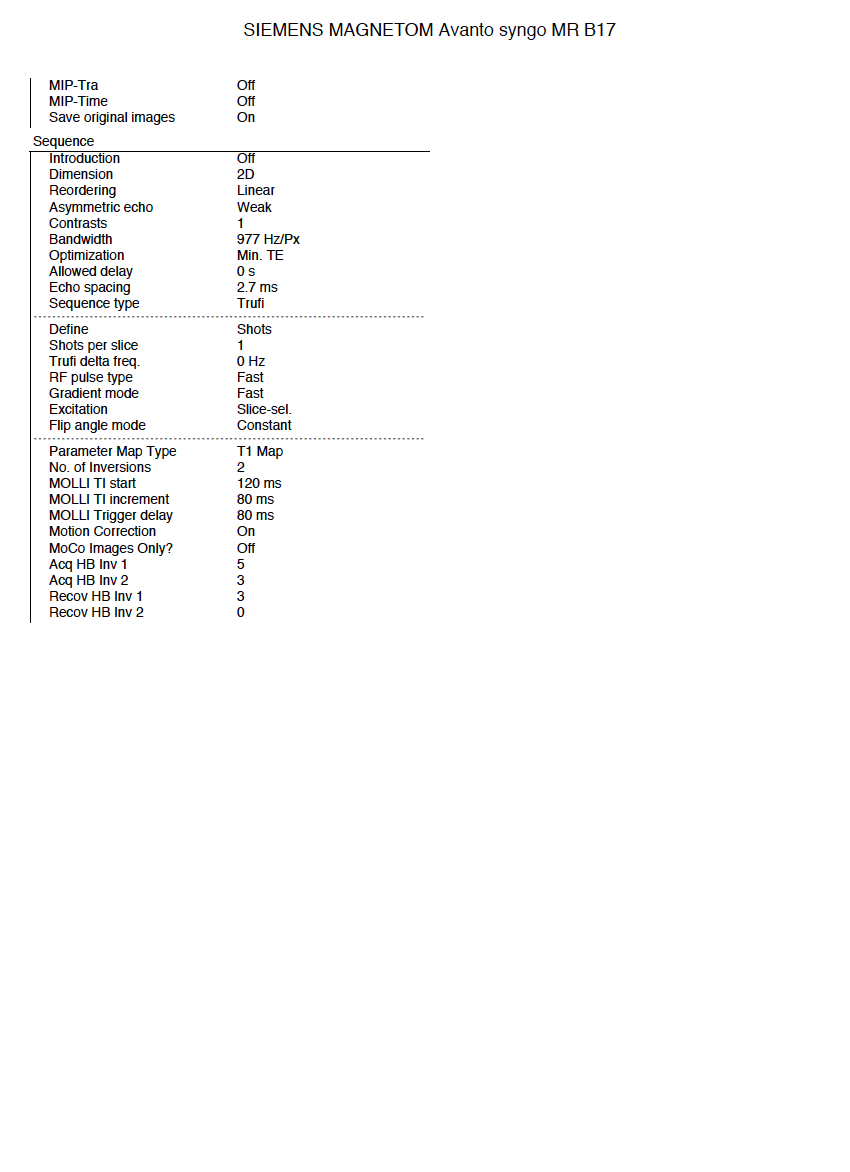


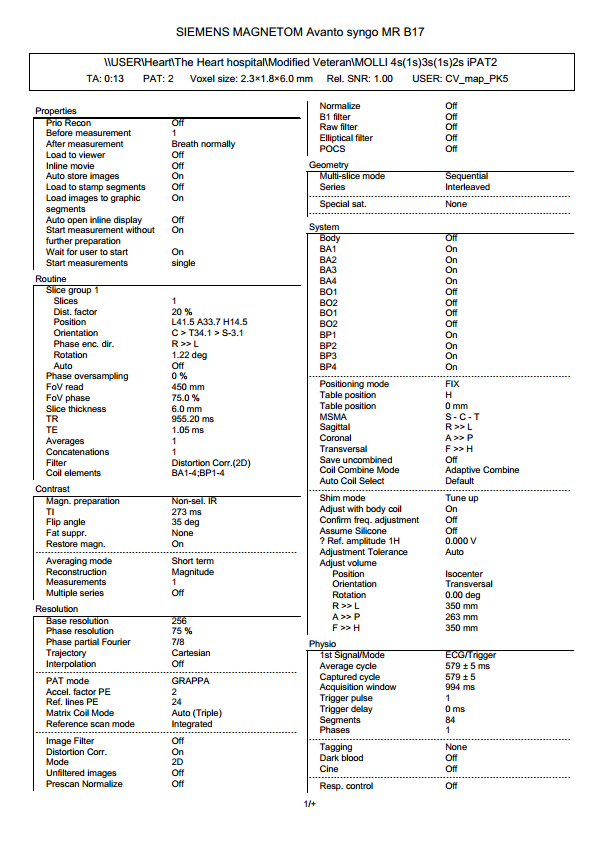


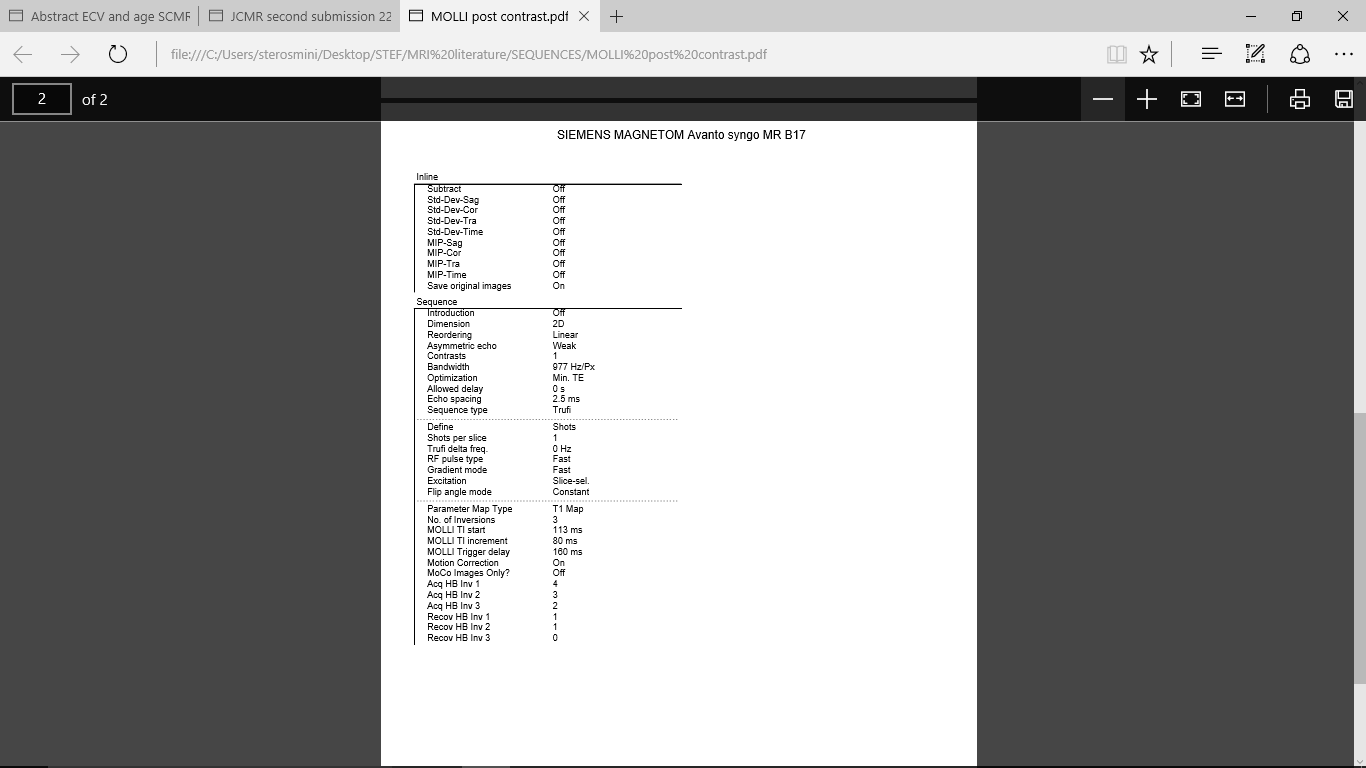


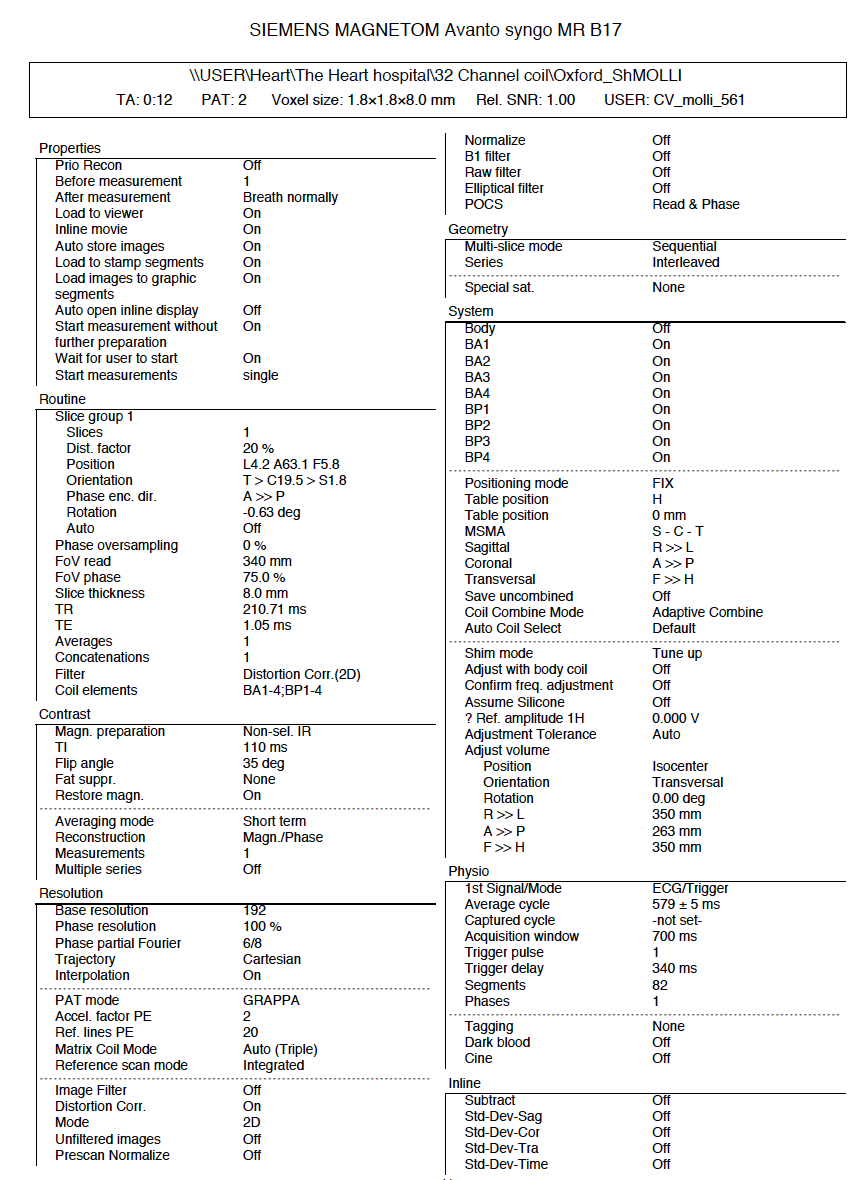


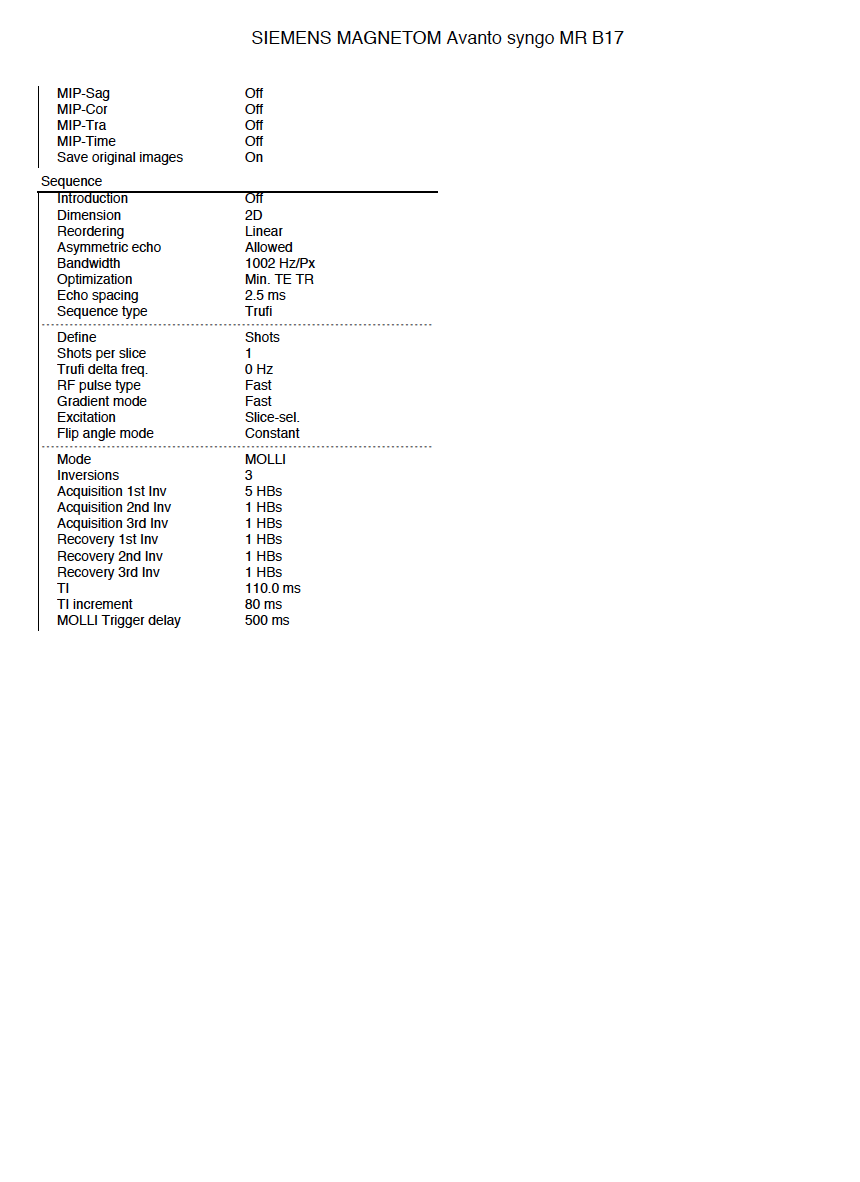


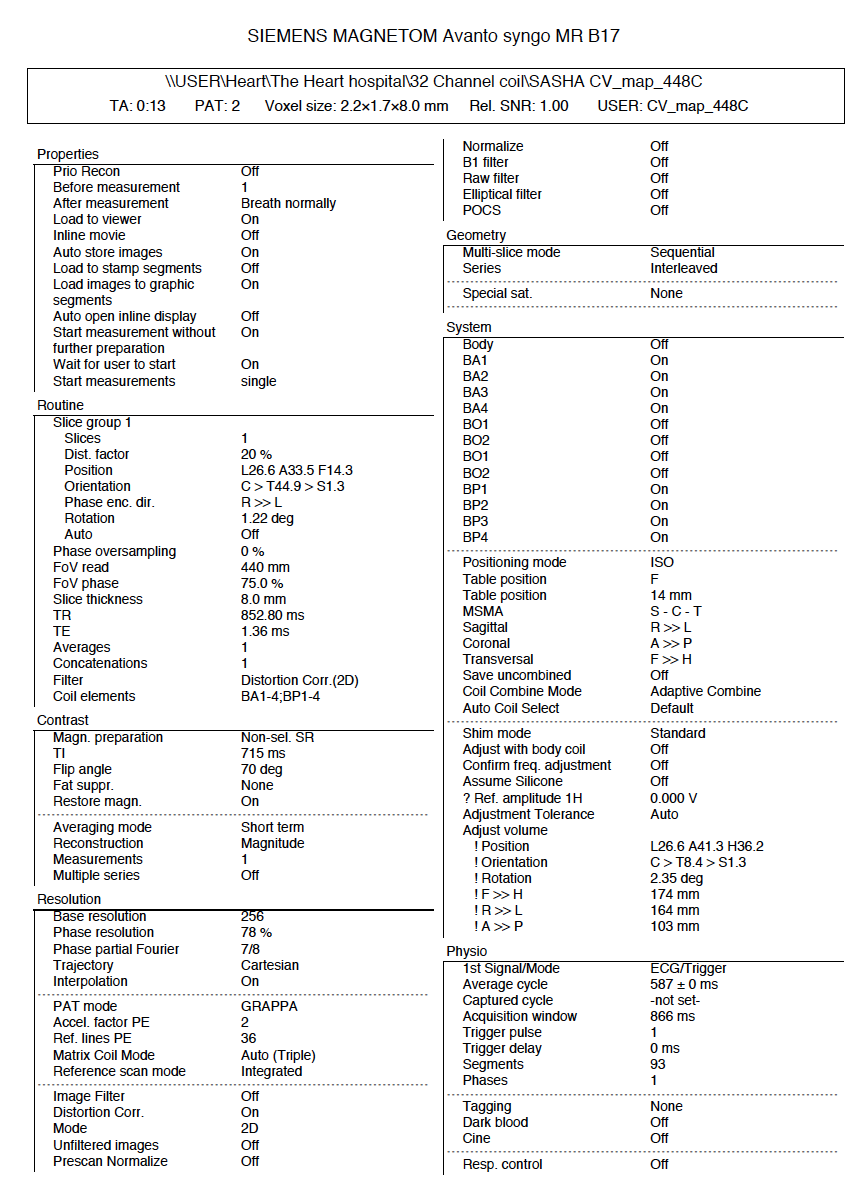


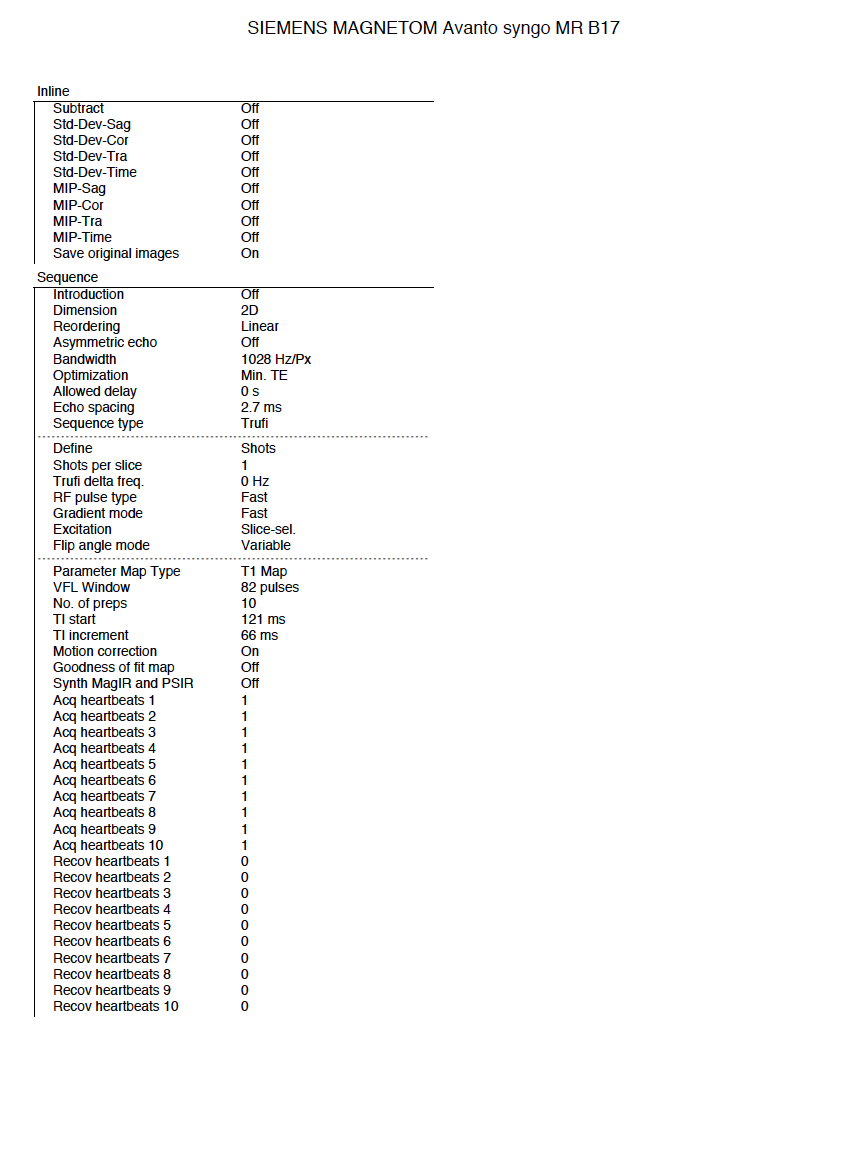

Supplement: Supplementary Data [file jey034_supplementary_data_27.04.17.docx]
